# *Faecalibacterium prausnitzii* promotes intestinal epithelial IL-18 production through activation of the HIF1α pathway

**DOI:** 10.3389/fmicb.2023.1298304

**Published:** 2023-12-14

**Authors:** Raphael R. Fagundes, Gabriela Bravo-Ruiseco, Shixian Hu, Sarah J. Kierans, Rinse K. Weersma, Cormac T. Taylor, Gerard Dijkstra, Hermie J. M. Harmsen, Klaas Nico Faber

**Affiliations:** ^1^Department of Gastroenterology and Hepatology, University of Groningen, University Medical Center Groningen, Groningen, Netherlands; ^2^Department of Medical Microbiology and Infection Prevention, University of Groningen, University Medical Center Groningen, Groningen, Netherlands; ^3^School of Medicine and Medical Science and the Conway Institute, University College Dublin, Dublin, Ireland

**Keywords:** HIF1α, IL-18, intestine, epithelial-bacteria interaction, HoxBan *in vitro* coculture system, *Faecalibacterium*, IBD

## Abstract

**Introduction:**

Intestinal epithelial cells produce interleukin-18 (IL-18), a key factor in promoting epithelial barrier integrity. Here, we analyzed the potential role of gut bacteria and the hypoxia-inducible factor 1α (HIF1α) pathway in regulating mucosal *IL18* expression in inflammatory bowel disease (IBD).

**Methods:**

Mucosal samples from patients with IBD (*n* = 760) were analyzed for bacterial composition, *IL18* levels and HIF1α pathway activation. Wild-type Caco-2 and CRISPR/Cas9-engineered Caco-2-*HIF1A*-null cells were cocultured with *Faecalibacterium prausnitzii* in a “Human oxygen-Bacteria anaerobic” *in vitro* system and analyzed by RNA sequencing.

**Results:**

Mucosal *IL18* mRNA levels correlated positively with the abundance of mucosal-associated butyrate-producing bacteria, in particular *F. prausnitzii*, and with HIF1α pathway activation in patients with IBD. HIF1α-mediated expression of *IL18*, either by a pharmacological agonist (dimethyloxallyl glycine) or *F. prausnitzii*, was abrogated in Caco-2-*HIF1A*-null cells.

**Conclusion:**

Butyrate-producing gut bacteria like *F. prausnitzii* regulate mucosal *IL18* expression in a HIF1α-dependent manner that may aid in mucosal healing in IBD.

## 1 Introduction

The human intestinal lumen harbors a complex ecosystem of microbial species, collectively named microbiota, that play a crucial role in gut homeostasis and are increasingly recognized as controllers of human health and disease (Manor et al., 2020). The intestinal epithelium is the barrier between the gut microbiota and the surrounding tissue. Interactions between the luminal bacteria and the adjacent epithelium control the homeostatic balance between the host and intestinal microbes. Disturbances in the composition of the microbiota are collectively named dysbiosis (Schippa and Conte, 2014; Ananthakrishnan, 2015) and have been associated with a variety of gastrointestinal and metabolic diseases, including type-2 diabetes, colon carcinoma and inflammatory bowel diseases (IBD) (Kostic et al., 2014; Schippa and Conte, 2014; Vich Vila et al., 2018). Such diseases are typically associated with a reduction in the dominant commensal *Faecalibacterium prausnitzii,* which is a strict anaerobic bacterium and a prominent producer of the short-chain fatty acid butyrate (Sokol et al., 2008; Cao et al., 2014).

The intestinal epithelium is in a constant state of physiological hypoxia due to its proximity to the anaerobic gut lumen that, in turn, drives the activation of the hypoxia-inducible factors 1α and 2α (HIF1α/HIF2α) (Fagundes and Taylor, 2017). HIF1α activation in the intestine transcriptionally promotes expression of genes encoding proteins that are involved in metabolic processes, barrier function and immune cell regulation, playing a key role in controlling intestinal homeostasis (Colgan et al., 2020). Under normoxic conditions, HIF1α protein is repressed and rapidly degraded through the action of asparaginyl- and prolyl-hydroxylases (FIH and PHD1-3, respectively) that utilizes oxygen as a cofactor for the hydroxylation of HIF1α. The activity of these hydroxylases is impaired during hypoxia, thereby preventing HIF1α repression and degradation that then translocate to the nucleus to activate gene transcription. Intestinal inflammation, characteristic in IBD, additionally creates a pathophysiological state of hypoxia that further activates HIF1α signaling to restore intestinal homeostasis (Colgan and Taylor, 2010; Cummins et al., 2016).

A variety of pro- and anti-inflammatory cytokines are produced to fight inflammation and promote tissue regeneration. Several cytokines are produced and secreted by the intestinal epithelium itself (Mahapatro et al., 2021). Interleukin-18 (IL-18; previously known as interferon-γ activating factor) is such a cytokine expressed by intestinal epithelial cells that stimulates epithelial barrier integrity, repair and tolerance of neighboring immune cells in the intestinal mucosa, thus acting as an anti-inflammatory cytokine (Zaki et al., 2010; Elinav et al., 2011; Normand et al., 2011; Mantovani et al., 2019; Yasuda et al., 2019). Balancing mucosal IL-18 levels is, however, of crucial importance, as it is a pro-inflammatory factor at sites of inflammation (Nakamura et al., 1989; Okamura et al., 1995; Tsutsumi et al., 2019). IL-18 is an IL-1-type cytokine produced as pro-IL-18 and processed intracellularly by the nucleotide-binding oligomerization domain (NLRP) inflammasome prior to secretion (Sellin et al., 2015). The NLRP inflammasome is composed of three subunits: the NLRP3 (or 6) protein as a platform, the adaptor protein ASC (apoptosis-associated speck-like protein containing a CARD) and CASP1 (Caspase-1) as the effector/proteolytic domain. Mice deficient in either NLRP3/6, CASP1 or IL-18 show exacerbated response to DSS-induced colitis (Zaki et al., 2010). Moreover, single nucleotide polymorphisms (SNPs) in the IL-18 receptor (*IL18R1*) and accessory protein (*IL18RAP*) loci are associated with both adult and severe early-onset IBD (Kim et al., 2014; Andreoletti et al., 2015; Soderquest et al., 2017). On the other hand, plasma levels IL-18 are elevated in ulcerative colitis patients (Wiercinska-Drapalo et al., 2005); and murine models revealed a paradoxical role for IL-18 in colitis, depending on disease stage (Nowarski et al., 2015; Pu et al., 2019). Interestingly, IL-18 was also shown to affect the gut microbiome composition directly (Levy et al., 2015; Liu et al., 2018).

We hypothesize that HIF1α transcriptional activation regulates the intestinal epithelial IL-18 production, stimulated by butyrate-producing bacteria and a potential target to promote mucosal healing in IBD. Here, we investigated the causal and functional connection between epithelial *IL18* expression, gut bacteria and HIF1α activation.

## 2 Materials and methods

### 2.1 Study population and ethical considerations

All patients included in this study were recruited by the IBD center of the of the Department of Gastroenterology and Hepatology of the University Medical Center Groningen (UMCG). Patients consented to participate in the 1000IBD project and Dutch parelsnoer IBD Biobank (Spekhorst et al., 2017; Imhann et al., 2019), and were at least 18-years old and had an established pre-existing diagnosis of IBD for at least 1 year. The study was approved by the Institutional Review Board (IRB) of the University Medical Center Groningen (UMC Groningen) (in Dutch: “Medisch Ethische Toetsingscommissie,” METc; IRB no. 2008/338 and 2016/424). The study was performed in accordance with the principles of the Declaration of Helsinki (2013). Diagnosis was based upon clinical, laboratory, endoscopic and histopathological criteria, the latter criteria also used for determining the inflammatory status of collected tissues (Lennard-Jones, 1989).

### 2.2 Intestinal biopsies collection

Intestinal biopsies were collected within the context of the 1000IBD project and Dutch parelsnoer IBD Biobank at time of colonoscopy procedures, which were part of standard clinical care. Intestinal biopsies were snap-frozen in liquid nitrogen shortly after the colonoscopy procedure. Biopsies were stored at −80°C until further processing. In total, 760 intestinal biopsies from 371 patients with IBD were analyzed in this study. During the endoscopic procedure, the inflammatory status of the biopsy was macroscopically categorized by assessing the mucosal morphology. Macroscopic inflammation was characterized by redness and edema, with or without ulceration of the intestinal mucosa. Additionally, histopathological evaluation later confirmed the macroscopic inflammation status.

### 2.3 DNA/RNA isolation and RNA sequencing

Isolation of DNA and RNA was performed using the AllPrep DNA/RNA mini kit (Qiagen; reference number: 80204) according to the manufacturer’s protocol. Homogenization of intestinal biopsies was performed in RLT lysis buffer including β-mercaptoethanol using the Qiagen Tissue Lyser with stainless steel beads (diameter of 5 mm; reference number: 69989). Sample preparation was performed using the BioScientific NextFlex mRNA sample preparation kit. RNA sequencing and data processing were performed as described earlier (Hu et al., 2021). Briefly, sequencing was executed with the Illumina NextSeq500 sequencer. Sampling and sequencing were performed in two different batches. RNA samples were pseudo-randomized on plates to mitigate potential batch effects. On average, 25 million reads were generated per sample. The quality of the raw reads was checked using FastQC at default parameters (ref v0.11.7). The adaptors identified by FastQC were clipped using Cutadapt (ref v1.1) with default settings. Sickle (ref v1.200) was used to trim low-quality ends from the reads (length < 25 nucleotides, quality < 20). Reads were aligned to the human genome (human_g1k_v37) using HISAT (ref v0.1.6) (2 mismatches allowed) and read sorting was done by SAMtools (ref v0.1.19). SAMtools flagstat and Picard tools (ref v2.9.0) were used to obtain mapping statistics. Seven samples with low percentage read alignment (<90%) were removed. Finally, gene expression was estimated through HTSeq (ref 0.9.1) based on Ensemble version 75 annotation. Gene-level expression data were normalized using a trimmed mean of M values, and log2 normalization was applied. From the RNA sequencing data, expression levels of *IL18, HIF1A*, *HIF2A*, HIF-hydroxylases (*EGLN1*, *EGLN2* and *EGLN3*), *VHL* and *HIF1AN* were selected for analysis. In addition, HIF1α and HIF2α scores (quotients calculated using the mRNA levels of either HIF-1α or HIF-2α divided by the sum total of the negative regulators of these factors), were calculating as described before (Brown et al., 2020; Fagundes et al., 2022).

### 2.4 16S rRNA gene sequencing

Biopsy-adherent bacteria abundances was assessed by 16S rRNA sequencing of human intestinal biopsies. Amplification of DNA extracted from biopsies was performed by PCR using modified 341F and 806R primers with a six-nucleotide barcode on the 806R primer on V3-V4 hypervariable region of the 16S rRNA gene. Then it was subjected to Illumina MiSeq paired-end sequencing as described previously (Swarte et al., 2020). The raw reads were trimmed and filtered using *Trimmomatic* v0.33 to obtain an average quality of 25 and a minimum length of 50 bases. Taxonomic assignment was following the pipeline https://benjjneb.github.io/dada2/. *Dada2* R package (v1.03) was used to get the amplicon sequence variants (ASVs). ASVs that were not present in 10% of all samples were filtered out. The rest were classified against SILVA database (release 132). The taxa with present rate > 10% was kept and centered log-ratio (clr) transformed for further analysis.

### 2.5 Human cell culture and CRISPR/Cas9 editing

Human epithelial colon adenocarcinoma cells (Caco-2, male, ATCC^®^, HTB-37TM, Manassas, United States) tested negative for mycoplasma infection and were used as *in vitro* representatives of intestinal epithelial cells. Single guide RNAs for *HIF1A* (sequence 5′-GATGGTAAGCCTCATCACAG-3′) were designed via Benchling^©^ online platform (https://www.benchling.com/) and cloned in pLenti-sgRNA backbone (Addgene, 71409). For lentivirus production, HEK293T cells in T25 were transfected using 15 μL Fugene HD (Promega, E2311), 250 μL Opti-MEM, 1.5 μg Lenti-iCas9-neo (Addgene, 85400) or pLenti-sgRNA (see above), 1 μg pMD2.G (Addgene, 12259) and 2.5 μg psPAX2 (Addgene 12260). Medium was refreshed 24 h after transfection and changed for Glutamax^™^ Dulbecco’s Modified Eagle Medium (DMEM, ThermoFisher Scientific Inc), supplemented with 10% fetal calf serum (FCS, Invitrogen), 1% non-essential amino acids (NEAA, Gibco) and 1% PSF antibiotic cocktail [penicillin (10 U/mL), streptomycin sulfate (100 μg/mL) and fungi zone; Lonza, Bazel, Switzerland]. Lentivirus suspension was collected 48 h after transfection, filtered through a 0.45 μm filter, aliquoted and stored at −80°C.

For generating stable Caco-2-iCas9 cell line, Caco-2 cells were seeded in a 6-well plate and incubated with 1 mL lentivirus suspension, supplemented with 8 μg/mL polybrene (Sigma-Aldrich, TR-1003). Cells were washed 24 h after infection with PBS and selection was started with Neomycin 2 mg/mL for 7 days with one cell passage in between. For introducing sgRNA, Caco-2-iCas9 cells were seeded in a 6-well plate and incubated with 1 mL lentivirus containing sgRNA (empty vector or against *HIF1A* – described above), supplemented with 8 μg/mL polybrene. Cells were washed 24 h after infection with PBS and selection was started 20 μg/mL puromycin for 7 days. Once puromycin treatment was finished, the resulting mutant Caco-2 cell line was cultured at low density on Glutamax™ DMEM, as described above. Twenty one clones were picked from the culture dish and cultured individually. Clones were expanded and DNA, RNA and protein collected for sequencing and functional validation of stable transfection. Treatments with control or Dimethyloxalylglycine (DMOG; D3695, Sigma Aldrich), at the concentrations of 1 or 5 mM, were carried out on selected clones for 24 h, followed by RNA isolation using TRIzol method, cDNA synthesis and gene expression analysis by RT-PCR, as described by Sadaghian Sadabad et al. (2015). Sequences for primers and probes are shown on Supplementary Table S1.

### 2.6 *Gaussia* luciferase assay

A HIF1α-responsive element (HRE)-*Gaussia* Luciferase reporter system (Cavadas et al., 2018) was used to assess HIF1α functional activity in Caco-2-*HIF1A-*null cells and empty vector controls. Caco-2 cells were transfected with 1 mg of the HRE-*Gaussia* Luciferase reporter construct in antibiotic-free media using Lipofectamine 2000 for 24 h, after which cells were treated with 1 mM DMOG for up to 48 h. Secreted luciferase was indicative of HRE-activity. Bioluminescence was quantified in media samples using the Pierce^™^ *Gaussia* Luciferase Glow Assay Kit (Thermo Scientific, 16161), carried out in a 96-well plate as per manufacturer’s instructions. Luminescence was determined using the CLARIOstar microplate reader (BMG Labtech, Ortenberg, Germany). Relative Luminescence Units (RLU) are presented normalized to total protein content (μg) for comparison of HIF1α functional activity levels.

### 2.7 Bacterial culture and HoxBan coculture system

HoxBan coculturing was performed essentially as described before (Sadaghian Sadabad et al., 2015): anaerobically-grown *F. prausnitzii* strain A2-165 (provided by S. Duncan, Aberdeen, UK) was inoculated in liquid broth containing yeast extract, casitone, and fatty acids and supplemented with 25 mM glucose as carbon and energy source (YCFAG). One (1) ml of an overnight *F. prausnitzii* culture was used to inoculate 1 liter of autoclaved agar (1.0%)-based-YCFAG (pH = 6.5) and cooled-down to approximately 40°C. In an anaerobic cabinet, 40 mL of this mixture was added to a 50 mL Falcon test tube and the bacteria-containing YCFAG-agar was allowed to solidify. After transfer to a laminar flow cabinet, 10 mL of 37°C -pre-warmed PSF-free DMEM medium was added to each tube. Next, the Caco-2 cells (Caco-2-*HIF1A*-null or Caco-2-empty vector) pre-grown on coverslips were laid upside-down on the top of the bacteria-containing YCFAG agar medium. The screw caps of the Falcon tubes were kept loosely tightened, to allow oxygen entry into the system for the Caco-2 cells. Caco-2/*F. prausnitzii* coculturing took place for 18 h at 37°C and 5% CO_2_, after which coverslips were collected and processed for downstream analyses.

### 2.8 RNA sequencing of cultured cell lines

Isolation of RNA from Caco-2 cells after HoxBan (co)culturing was performed using the RNeasy Plus Mini Kit (Qiagen; reference number: 74134) according to the manufacturer’s protocol. Homogenization of cells was performed in RLT lysis buffer containing β-mercaptoethanol. Sample preparation was performed using the QuantSeq 3’mRNA-Seq Library Prep Kit sample preparation kit (Lexogen; reference number 015.95). RNA sequencing was executed with the Illumina NextSeq500 sequencer. RNA samples were pseudo-randomized on plates.

### 2.9 Statistics

The associations between gene expressions and microbial taxa in 760 intestinal biopsies from 371 IBD patients were assessed using mixed linear models adjusting for batch, age, sex, BMI, smoking, tissue location and inflammation status. Patient IDs were included as random effect to account for repeat measurements. Data were presented as mean ± standard deviation (SD). Assessment of normality was performed using histograms and normal probability plots (Q-Q plots). Differential gene expression analysis between groups was analyzed using independent sample *t*-tests with the EdgeR package on RStudio (version 1.4.1717; Rstudio, Boston, MA, United States) or ordinary One-way ANOVA using GraphPad Prism 9.0 (GraphPad software, San Diego, CA, United States). Gene expression was normalized using the trimmed mean of M-values normalization method (TMM) and then 2log-transformation was applied. Finally, gene expression means were centered to zero and standard deviations scaled to one. Data visualization was performed using GraphPad Prism 9.0 and Rstudio. Two-tailed *p*-values ≤ 0.05 were considered statistically significant.

## 3 Results

### 3.1 Gene expression levels of *IL18* correlate with bacterial abundance in the intestinal mucosa of IBD patients

**Figure 1:**
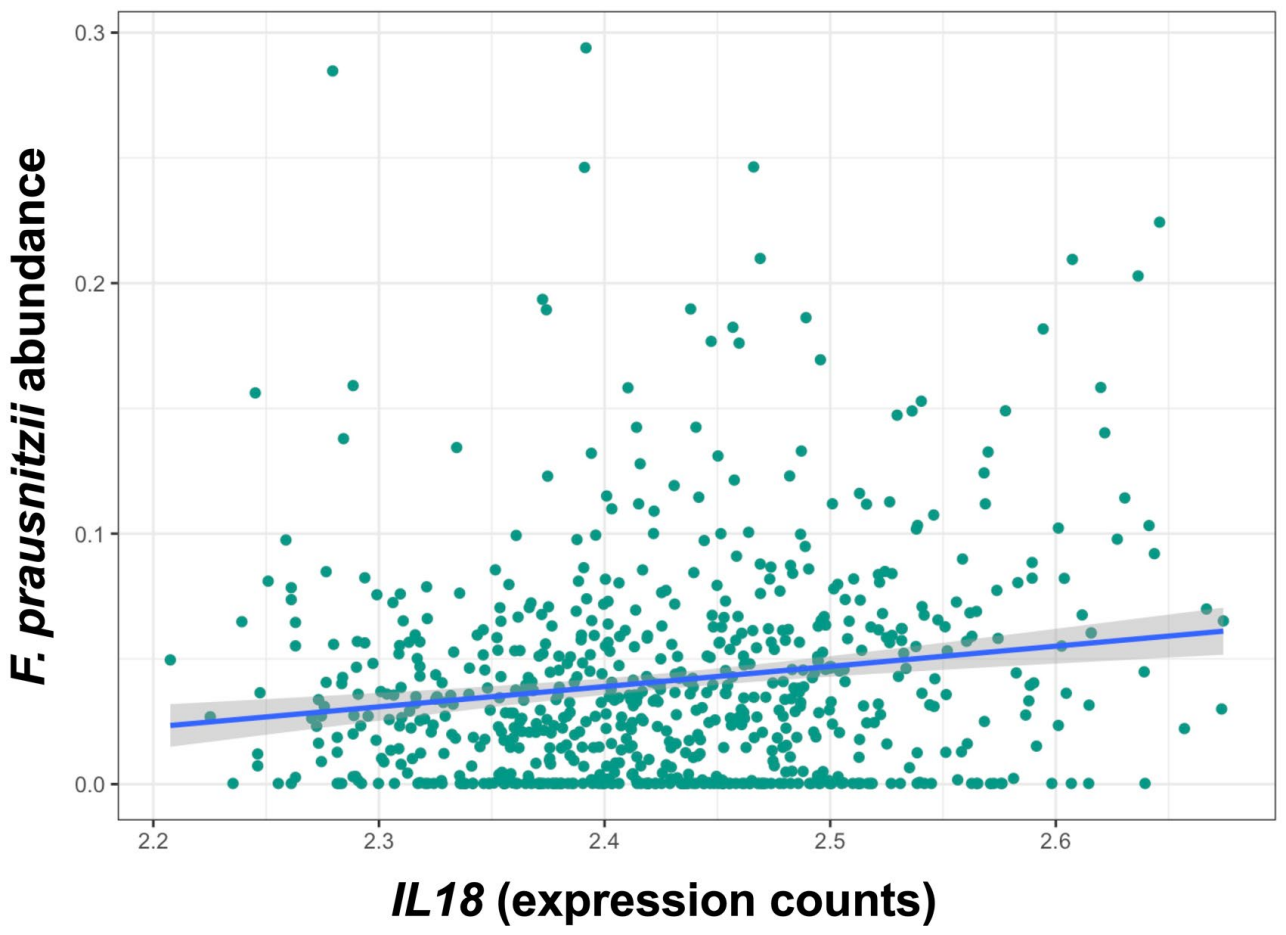
*IL18* expression levels are significantly associated with abundances of *F. prausnitzii* in human intestinal mucosa. *F. prausnitzii* abundance is positively correlated to *IL18* expression in intestinal mucosa. Patient cohort study performed in *N* = 760 biopsies.

Descriptive statistics of the patient cohort is described in Table 1. Mucosal *IL18* expression in 760 intestinal mucosa biopsies from 371 IBD patients was obtained from an in-house dataset of RNA sequencing dataset (Hu et al., 2022) and was correlated to the abundance of bacterial genera, as established by 16S rRNA gene sequencing of the same biopsies. A significant positive correlation between *IL18* expression and mucosal levels of prominent butyrate-producing bacteria was observed, including *Ruminococcaceae*, *Ruminococcus* species, *Agathobacter*, and *Faecalibacterium* (Table 2). In contrast, *IL18* expression correlated negatively with *GCA-900066575, Sutterella* and *Bacteroides* abundances (Table 2). Species from the *Sutterella* genus have been described as potential pathogens associated with inflammatory diseases (Gryaznova et al., 2021). At the species level, a significant positive correlation was observed between *IL18* expression and *F. prausnitzii* (Figure 1, effect size = 0.227, *p* = 7.18 × 10^−4^). While no significant differences were observed in mucosal-associated *F. prausnitzii* abundances between inflamed and non-inflamed tissue (Table 3), we identified correlations between the abundance of *F. prausnitzii* and certain bacterial genera outlined in Table 2 (Supplementary Figure S2). Given the positive association of *IL18* expression with butyrate-producing bacteria, and since butyrate activates the epithelial HIF1α pathway (Kelly et al., 2015), we next investigated whether mucosal *IL18* expression correlates with HIF1α pathway activation.

**Table 1:** Descriptive statistics of cohort of IBD patients (*n* = 371), segregated by disease subtype.

Variable	CD	UC
Individual (patient) level	*n* = 202	*n* = 169
Age (years)	40.4 ± 15.2	44.1 ± 15.4
**Sex, *n* (%)**		
Female	132 (65.3)	81 (47.9)
BMI (kg/m^2^)	25.4 ± 4.7	26.2 ± 4.3
**Current smoking, *n* (%)**		
Yes	61 (30)	22 (13.1)
**Montreal age (A), *n***		
A1 (≤16 years)	31	12
A2 (17–40 years)	135	103
A3 (>40 years)	35	54
**Montreal location (L), *n***		–
L1 (ileal disease)	44	–
L2 (colonic disease)	35	–
L3 (ileocolonic disease)	91	–
L1 + L4	6	–
L2 + L4	3	–
L3 + L4	14	–
**Montreal behavior (B), *n***		–
B1 (non-structuring, non-penetrating)	80	–
B2 (structuring)	30	–
B3 (penetrating)	17	–
B1 + P (perianal disease)	28	–
B2 + P (perianal disease)	28	–
B3 + P (perianal disease)	10	–
**Montreal extension (E), *n***	–	
E1 (proctitis)	–	11
E2 (left-sided colitis)	–	47
E3 (pancolitis)	–	90
**Montreal severity (S), *n***	–	
S0 (remission)	–	7
S1 (mild)	–	16
S2 (moderate)	–	68
S3 (severe)	–	35
**Medication use**		
Aminosalicylates, *n*	23	29
Thiopurines, *n*	74	58
Steroids, *n*	69	74
Methotrexate, *n*	18	4
TNF-α-antagonists, *n*	48	12

CD, Crohn’s disease; UC, ulcerative colitis.

**Table 2:** Bacteria significantly correlated with *IL18* expression in intestinal mucosa of IBD patients ordered by significance.

Bacteria (genera)	Effect size	Standard error	*p*-value
**Positive associations**			
*Ruminococcaceae_UCG.005*	3.648	0.992	0.0003
*Ruminococcus_1*	5.775	1.635	0.0004
*Agathobacter*	0.503	0.141	0.0004
*Faecalibacterium*	0.227	0.067	0.0007
*Escherichia/shigella*	0.283	0.099	0.0042
*Lachnospiraceae_ND3007_group*	4.073	1.677	0.0154
*Ruminococcaceae_UCG.013*	7.997	3.334	0.0167
*Haemophilus*	0.222	0.095	0.0191
*Ruminococcus_2*	1.832	0.862	0.0338
*Collinsella*	13.888	6.926	0.0453
*Blautia*	0.506	0.257	0.0497
**Negative associations**			
*Bacteroides*	−0.088	0.027	0.0013
*GCA.900066575*	−2.967	0.962	0.0021
*Sutterella*	−0.219	0.091	0.0166

**Table 3:** Comparison between *F. prausnitzii* abundances between inflamed and non-inflamed mucosa tissue of IBD patients.

Sample origin	*p*-value
**Crohn’s disease**	
Ileum	0.94
Colon	0.10
**Ulcerative colitis**	
Colon	0.28

### 3.2 *IL18* expression correlates with HIF1α activity in intestinal mucosa of IBD patients

**Figure 2:**
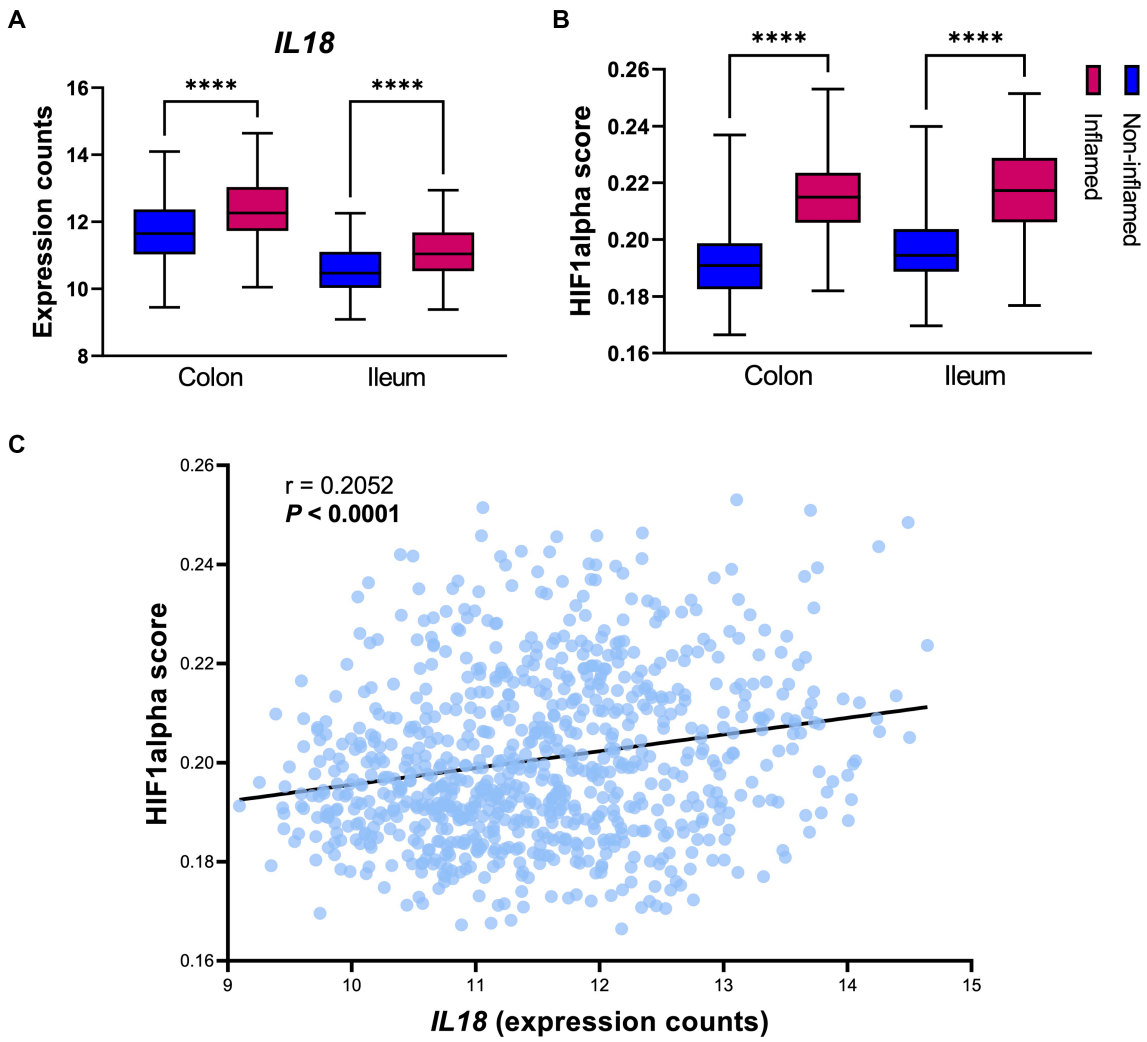
*IL18* expression strongly associates to HIF1α activation capacity in the human intestinal mucosa. **(A)** Gene expression levels of *IL18* in intestinal mucosa of IBD patients, divided by location (colon and ileum). **(B)** HIF1α activation capacity (calculated by HIF1α scores) in intestinal mucosa of IBD patients. **(C)** Spearman correlation analysis shows significant positive association between *IL18* and HIF1α scores in intestinal mucosa of IBD patients. Patient cohort study performed in *n* = 760 samples (biopsies), segregated by their inflammatory statues and biopsy location as colonic inflamed (*n* = 195), colonic non-inflamed (*n* = 307), ileal inflamed (*n* = 78) and ileal non-inflamed (*n* = 180) biopsies. Data presented as box and whiskers (min to max); ****p < 0.0001.

*IL18* mRNA levels were significantly enhanced in inflamed intestinal mucosa when compared to non-inflamed tissue, both in colon and in ileum (Figure 2A). Similarly, HIF1α scores [representing HIF1α activation capacity (Brown et al., 2020)] were increased in inflamed mucosa in both locations, when compared to non-inflamed mucosa (Figure 2B). In line, Spearman correlation analysis revealed a positive association between *IL18* and HIF1α scores in the intestinal mucosa of IBD patients (Figure 2C; *r* = 0.2052 and *p* < 0.0001).

### 3.3 *HIF1A* knock out in intestinal epithelial cells

**Figure 3:**
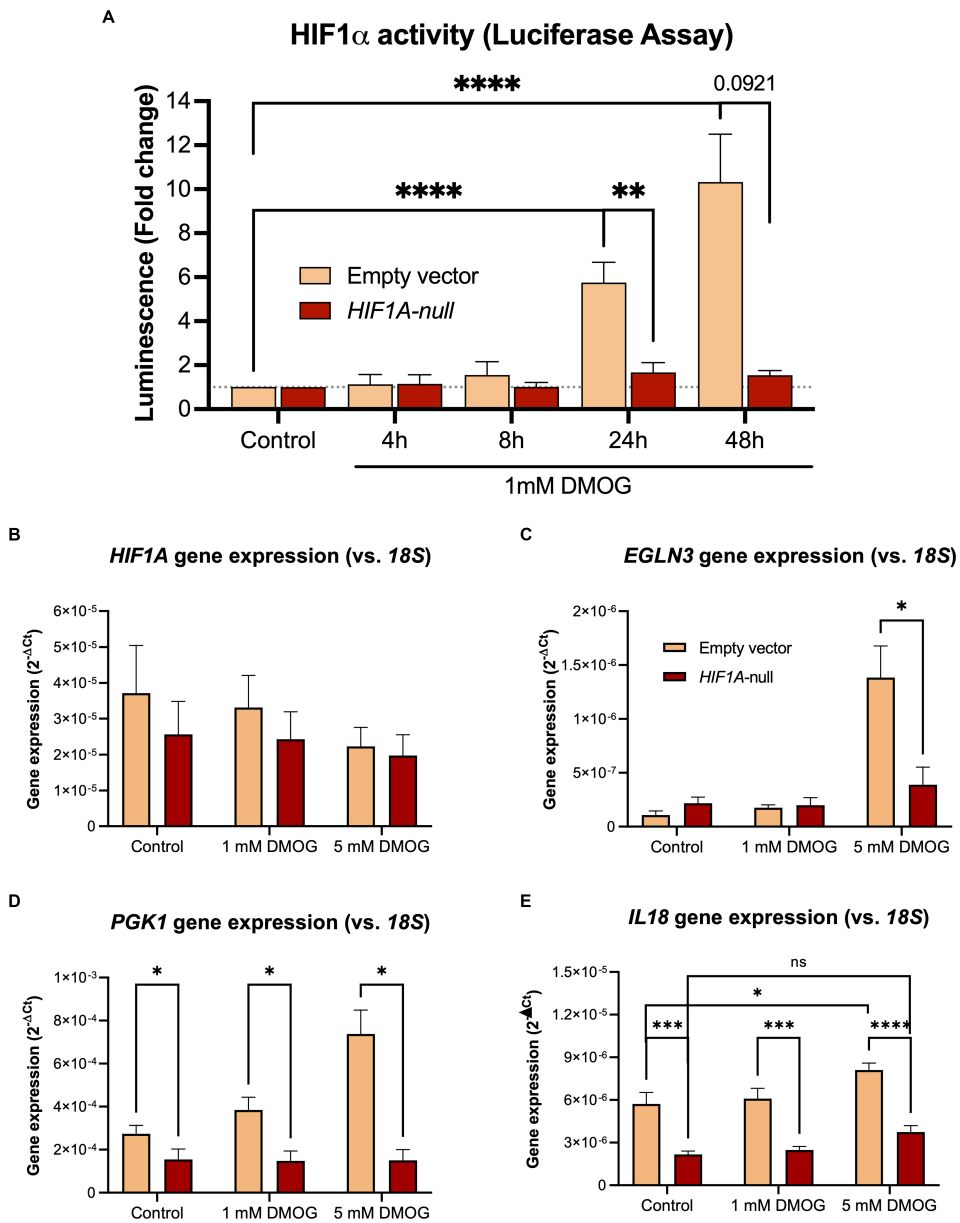
Stable knockout of *HIF1A* in Caco-2 cells leads to decreased HIF1α signaling upon pathway activation. **(A)** Time-dependent HIF1α responsive element (HRE)-luciferase assay over 48 h treatment with 1 mM DMOG. Gene expression levels of **(B)** *HIF1A* and the HIF1α pathway targets **(C)** *PGK1*, **(D)** *EGLN3*, and **(E)** *IL18* in Caco-2-*HIF1A*-null and control cells treated with 1 or 5 mM DMOG for 24 h. Data presented as mean ± SD. All experiments were performed with *n* = 3; *p < 0.05, **p < 0.01, ****p < 0.0001.

In order to establish whether *IL18* expression is under direct control of HIF1α, we inactivated the *HIF1A* gene in Caco-2 cells using CRISPR/Cas9 technology (Figure 3). We functionally tested the loss of HIF1α activation in the Caco-2-*HIF1A*-null cells using a secreted *Gaussia* luciferase driven by HIF1α responsive elements (HRE) over 48 h exposition to 1 mM of the prolyl-hydroxylase inhibitor DMOG (Figure 3A). We observed an almost complete loss of HIF1α response in Caco-2-HIF1A-null cells, compared to Caco-2(-iCas9-empty vector) control cells, which had HRE-luciferase signal up to 10-fold upregulated of over 48 h exposition to DMOG. Because of the mutation introduced by the guide RNA, *HIF1A* mRNA remained detectable in Caco-2-*HIF1A*-null cells, with no significant difference compared to Caco-2-empty vector control cells (Figure 3B). Pharmacological activation of HIF1α (using DMOG) dose-dependently enhanced mRNA levels of typical HIF1α target genes *EGLN3* and *PGK1* in Caco-2 control cells, which was blunted in the Caco-2-*HIF1A*-null cells (Figures 3C,D). Similarly, *IL18* expression was slightly, but significantly induced by DMOG in Caco-2 control cells, which was blocked in the absence of *HIF1A* (Figure 3E). Interestingly, basal *IL18* levels were already significantly lower in Caco-2*-HIF1A*-null cells compared to Caco-2 control cells, which was also observed for *PGK1* (Figures 3D,E, respectively).

### 3.4 *Faecalibacterium prausnitzii* regulates gene transcription in a HIF1α-dependent manner in intestinal epithelial cells

**Figure 4:**
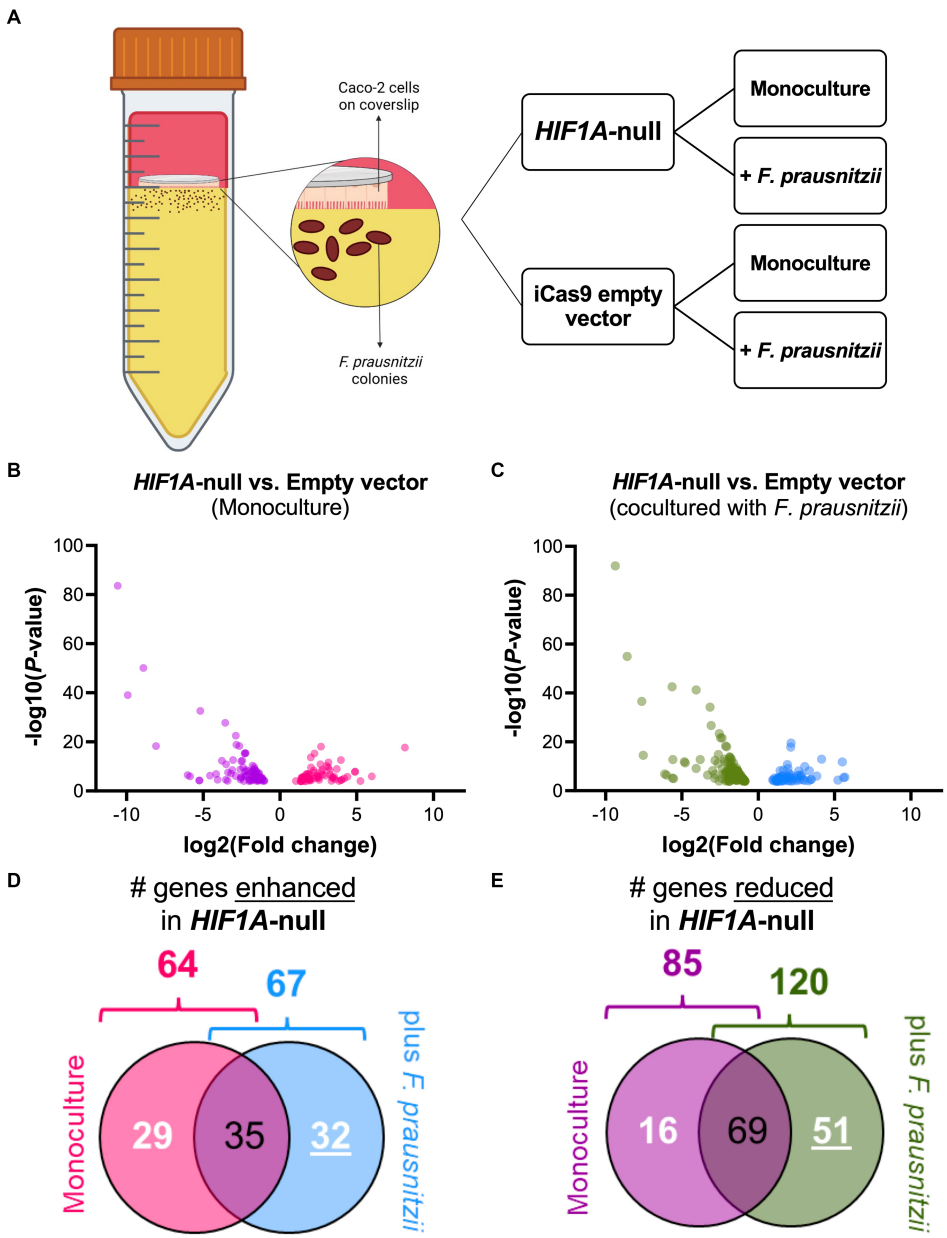
Differential gene expression (DGE) analysis of Caco-2-*HIF1A*-null upon coculture with *F. prausnitzii*, compared to Caco-2 empty vector controls. **(A)** Schematic representation of the HoxBan coculture system, including experiment layout. Caco-2-*HIF1A*-null cells, or appropriate control (iCas9 empty vector), were cultured in the HoxBan system for 18 h in the absence or presence of *F. prausnitzii* inoculum (in figure, mono and co, respectively). Volcano plots showing differential gene expression analysis comparing Caco-2-*HIF1A*-null cells and Caco-2-empty vector in monoculture (**B**; purple and pink dots represent downregulated and upregulated genes, respectively) and coculture with *F. prausnitzii* (**C**; green and blue dots represent downregulated and upregulated genes, respectively). All dots display significant observations with *p* < 0.05. Veen diagrams were used to discover uniquely **(D)** upregulated and **(E)** downregulated DGE (numbers underlined inside blue and green diagrams, respectively) in Caco-2-*HIF1A*-null cultured with *F. prausnitzii*, compared to Caco-2-empty vector in same condition. All experiments performed in *n* = 3.

To analyze the putative role of HIF1α in regulating *F. prausnitzii*-mediated transcriptional responses, Caco-2 control (empty vector) and Caco-2*-HIF1A*-null cells were cultured for 18 h in the absence and presence of the strict anaerobic bacterium *F. prausnitzii* using the “Human-oxygen Bacteria-anaerobic” (HoxBan) coculture system (schematically drawn in Figure 4A), followed by RNA sequencing of the Caco-2 cells. In the absence of *F. prausnitzii*, a total of 149 genes were significantly differentially expressed between Caco-2 control and Caco-2-*HIF1A*-null cells (Figure 4B), while 187 genes were significantly differentially expressed between those 2 cell lines in the presence of *F. prausnitzii* (Figure 4C). The absence of HIF1α significantly enhanced expression of 64 genes, and reduced levels of 85 genes (Figures 4D,E), when grown in the absence *F. prausnitzii* in the HoxBan setup. In the presence of *F. prausnitzii*, expression levels of 67 genes were enhanced, and of 120 genes reduced (Figures 4D,E) in the absence of HIF1α. Overlaying these effects, we observed enhanced expression of 32 genes (Table 4), and reduced expression of 51 genes (Table 5), specifically in *F. prausnitzii*-cocultured Caco-2-*HIF1A*-null cells, when compared to *F. prausnitzii*-cocultured Caco-2 control cells (Figures 4D,E). Among the 32 upregulated genes, two (*NRP2* and *CPT1A*) were previously reported to be suppressed by HIF1α signaling (Coma et al., 2011; Du et al., 2017; Tan and Welford, 2020; Ezzeddini et al., 2021), whereas no established HIF1α-upregulated targets were detected among these genes that were enhanced by *F. prausnitzii* in Caco-2-*HIF1A*-null cells. On the other hand, the 51 downregulated genes were enriched for known HIF1α targets, including *PFKB3* (Minchenko et al., 2002), *FOS* (Ebersole et al., 2018), *IER3* (Ravaud et al., 2015), *ANXA1* (Bizzarro et al., 2017), *HK2* (Mathupala et al., 2001), *KDM3A* (Mimura et al., 2012), *MT2A* (Mo et al., 2020), *LBH* (Jiang et al., 2019), *DDIT4* (Gharibi et al., 2016), and *TGM2* (Jang et al., 2010). Interestingly, *IL18* was among these 51 genes (Table 5), of which the expression appears to be controlled through *F. prausnitzii-*mediated activation of HIF1α.

**Table 4:** Uniquely upregulated genes in *F. prausnitzii*-cocultured Caco-2-*HIF1A*-null cells, compared to *F. prausnitzii*-cocultured Caco-2 control cells.

Gene name	Log2(Fold Change)	*p*-value	FDR
*SLC22A9*	5.22	4.79E-05	1.87E-02
*SLC39A2*	3.83	8.94E-07	6.40E-04
*LTK*	3.43	3.22E-05	1.34E-02
*AR*	3.29	8.78E-05	3.16E-02
*CYS1*	3.03	5.96E-05	2.25E-02
*DSC3*	2.92	2.68E-05	1.16E-02
*CCDC136*	2.52	7.35E-05	2.75E-02
*ZNF618*	2.39	5.71E-06	3.09E-03
*JAKMIP2*	2.21	1.15E-04	3.93E-02
*CCDC71*	2.04	8.71E-05	3.15E-02
*LGI4*	2.00	6.66E-08	6.45E-05
*THPO*	1.89	1.12E-05	5.32E-03
*USP51*	1.86	5.11E-06	2.84E-03
*PRAF2*	1.81	6.52E-06	3.40E-03
*ZNF813*	1.81	2.34E-05	1.04E-02
*GABBR1*	1.69	9.05E-05	3.24E-02
*ALG10B*	1.65	4.41E-05	1.74E-02
*CIBAR1*	1.63	1.08E-06	7.62E-04
*CPT1A*	1.55	7.99E-05	2.95E-02
*NCOA5*	1.51	1.26E-04	4.17E-02
*CRABP1*	1.43	5.86E-09	6.91E-06
*PGBD5*	1.38	2.58E-05	1.12E-02
*PPARGC1B*	1.37	2.90E-05	1.23E-02
*ADGRV1*	1.36	5.47E-06	2.99E-03
*SI*	1.29	7.49E-05	2.78E-02
*STOX1*	1.29	1.02E-04	3.56E-02
*DUSP23*	1.26	1.35E-04	4.41E-02
*NSMCE3*	1.22	2.03E-05	9.23E-03
*IFT74*	1.15	4.44E-05	1.74E-02
*CRYBG1*	1.12	1.20E-04	4.04E-02
*NRP2*	1.08	6.68E-06	3.43E-03
*CLSTN2*	0.99	6.72E-05	2.53E-02

**Table 5:** Uniquely downregulated genes in *F. prausnitzii*-cocultured Caco-2-*HIF1A*-null cells, compared to *F. prausnitzii*-cocultured Caco-2 control cells.

Gene name	Log2(Fold Change)	*p*-value	FDR
*JAM3*	−5.61	6.54E-06	3.40E-03
*AC107021.2*	−5.54	1.03E-05	5.04E-03
*ELFN1-AS1*	−3.31	4.15E-07	3.28E-04
*DDX60*	−2.56	1.00E-04	3.53E-02
*GALNT5*	−2.35	1.06E-05	5.14E-03
*ODAM*	−2.10	1.33E-08	1.43E-05
*DNAH6*	−2.03	5.75E-05	2.21E-02
*PLD5*	−1.92	1.19E-04	4.04E-02
*LIMCH1*	−1.91	1.39E-04	4.46E-02
*NRP1*	−1.90	5.96E-06	3.20E-03
*IFIT3*	−1.89	4.54E-07	3.50E-04
*SLC15A2*	−1.88	1.25E-04	4.17E-02
*STK39*	−1.75	2.43E-06	1.51E-03
*GLIPR1*	−1.75	2.85E-07	2.41E-04
ANXA1*	−1.62	9.51E-06	4.69E-03
*LINC00668*	−1.51	1.48E-06	9.94E-04
*SMPDL3B*	−1.44	5.85E-05	2.22E-02
PFKFB3*	−1.43	2.65E-06	1.60E-03
*UBE2L6*	−1.42	2.84E-05	1.21E-02
*SMC1B*	−1.42	5.79E-05	2.22E-02
*AC012615.1*	−1.41	2.48E-05	1.09E-02
*UPK1B*	−1.36	4.53E-06	2.59E-03
MT2A*	−1.34	6.50E-07	4.82E-04
DDIT4*	−1.32	1.32E-08	1.43E-05
*PLSCR1*	−1.31	2.12E-08	2.16E-05
*IL18*	−1.30	2.94E-06	1.75E-03
*COL12A1*	−1.30	3.81E-05	1.55E-02
*TFPI*	−1.26	4.67E-07	3.55E-04
*PARP9*	−1.26	2.98E-07	2.48E-04
TGM2*	−1.21	3.57E-06	2.10E-03
*SPTSSB*	−1.20	4.89E-06	2.75E-03
FOS*	−1.16	1.74E-06	1.13E-03
*FAM171B*	−1.14	5.45E-06	2.99E-03
HK2*	−1.11	1.26E-05	5.92E-03
*CITED2*	−1.07	6.56E-06	3.40E-03
LBH*	−1.05	3.13E-05	1.31E-02
*TM4SF5*	−1.04	1.07E-04	3.70E-02
*PPP3CA*	−1.03	7.43E-06	3.78E-03
*PRSS23*	−1.01	7.79E-06	3.90E-03
*CRIM1*	−0.97	1.78E-05	8.18E-03
KDM3A*	−0.97	2.98E-05	1.25E-02
*SP5*	−0.96	1.03E-04	3.59E-02
*CYSTM1*	−0.94	2.75E-05	1.18E-02
IER3*	−0.94	8.21E-05	2.99E-02
*LAMC2*	−0.93	9.67E-05	3.44E-02
*TTR*	−0.93	4.00E-05	1.60E-02
*MBNL3*	−0.92	4.87E-05	1.89E-02
*PARP14*	−0.91	1.22E-04	4.10E-02
*KRT8*	−0.89	1.02E-04	3.56E-02
*PEG10*	−0.88	1.32E-04	4.33E-02
*COTL1*	−0.86	1.36E-04	4.42E-02

*Known HIF1α targets.

### 3.5 *Faecalibacterium prausnitzii* enhances *IL18* expression in Caco-2 cells in a HIF1α-dependent manner

**Figure 5:**
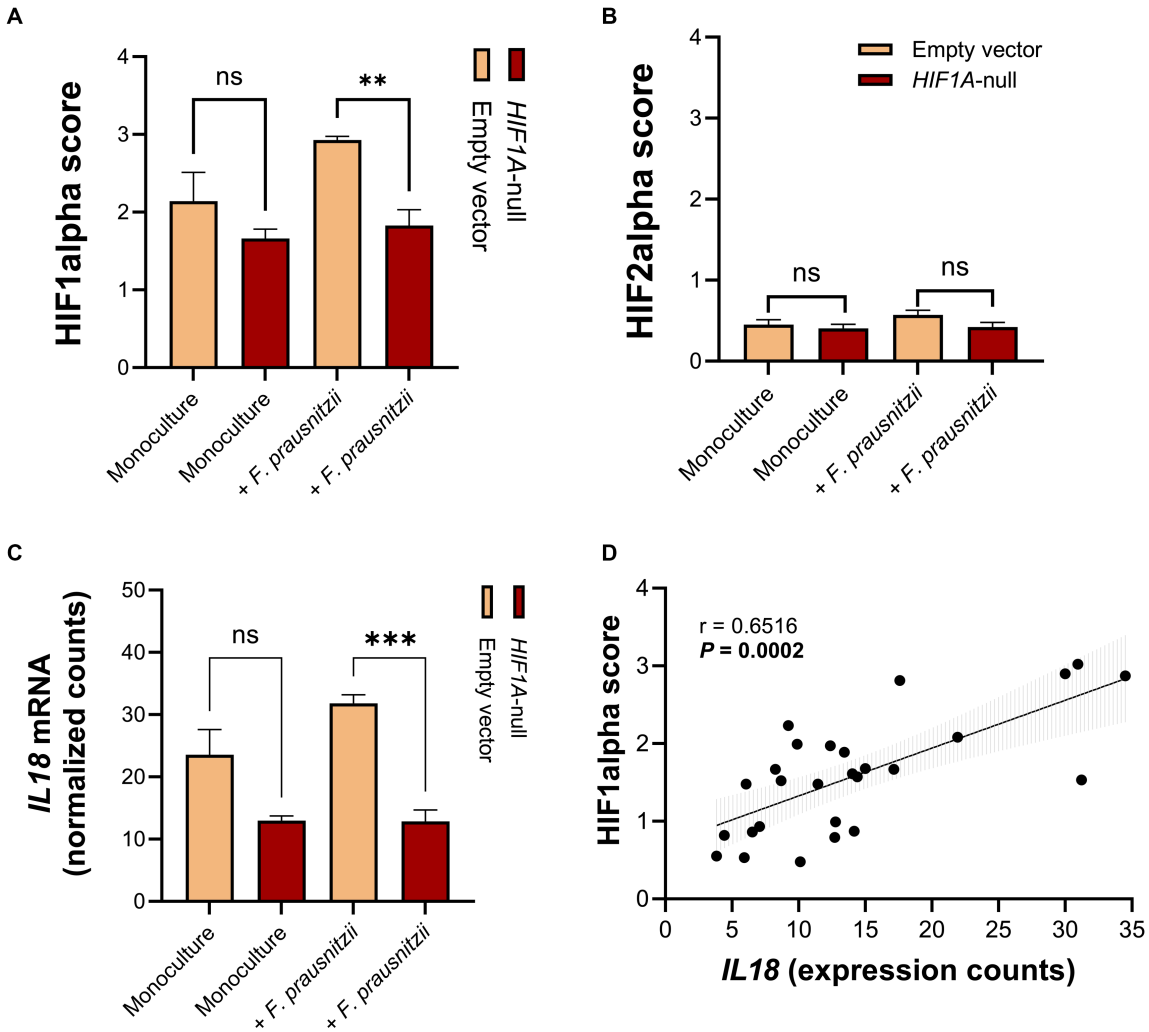
RNA sequencing analysis reveals that *HIF1A* ablation prevents upregulation of *IL18* by *F. prausnitzii* in intestinal epithelial cells. **(A,B)** HIF1α and HIF2α scores shows a decrease in HIF1α, but not HIF2α, activation capacity in Caco-2-*HIF1A*-null, compared to Caco-2-empty vector control in coculture with *F. prausnitzii*. **(C)** Normalized gene expression counts of *IL18* in Caco-2-*HIF1A*-null cells, compared to Caco-2-empty vector in monoculture and coculture with *F. prausnitzii*. **(D)** Spearman correlation analysis between *IL18* gene expression and HIF1α scores on Caco-2 cells in the HoxBan system. All experiments performed in *n* = 3 (ns = not significant, **p < 0.001 and ***p < 0.0001).

Specifically analyzing the HIF1α and -2α scores from the RNA sequencing results, we observed a (non-significant) enhancement of HIF1α scores in Caco-2 control cells when comparing monocultures (Caco-2 cells without *F. prausnitzii*) to Caco-2-*F. prausnitzii* cocultures (Figure 5A). *F. prausnitzii* did not enhance the HIF1α score in Caco-2-*HIF1A*-null cells (Figure 5A, red bars). Notably, the HIF1α score was significantly lower in Caco-2-*HIF1A*-null cells cocultured with *F. prausnitzii* when compared to similarly-cultured Caco-2 control cells (Figure 5A; *p* < 0.0001). HIF2α scores were lower than HIF1α scores in all conditions, with no significant changes between different conditions (Figure 5B). *IL18* mRNA levels exactly followed the HIF1α score: *F. prausnitzii* enhanced *IL18* levels non-significantly in Caco-2 control cells and were significantly lower in *F. prausnitzii*-cocultured Caco-2-*HIF1A*-null cells (Figure 5C, *p* < 0.001). In line, a highly significant positive correlation between *IL18* gene expression and HIF1α scores was observed (*r* = 0.6516 and *p* = 0.0002; Figure 5D). Taken together, these data point to a *F. prausnitzii*-mediated regulation of *IL18* in the intestinal epithelium that is controlled by HIF1α.

## 4 Discussion

In this study, we found that mucosal *IL18* expression associates positively with gut commensal and butyrate-producing bacteria in a cohort of 371 IBD patients, while it negatively associates with potential pathogens. At the bacterial strain level, mucosal *IL18* gene expression positively associated with the mucosal abundance of the gut commensal *F. prausnitzii*. Using the HoxBan *in vitro* coculture model, we showed that this mechanistic relationship is directly regulated by activation of the transcription factor HIF1α in intestinal epithelial cells. Moreover, we described transcriptional modulations that are triggered in a HIF1α-dependent manner by *F. prausnitzii* on intestinal epithelial cells, also involving the regulation of *IL18* expression.

In our study, we provided evidence that epithelial expression levels of *IL18* are regulated in a HIF1α-dependent manner and associates positively to bacterial species directly linked to epithelial health. However, the absence of protein quantification in our study represents a limitation, and further investigations may provide a more comprehensive understanding of the molecular mechanisms involved. Nonetheless, in line with our results, previous studies using germ-free mouse models showed a decrease in *IL18* gene expression and secretion, when compared to conventionally raised mice (Singh et al., 2014; Levy et al., 2015). Additionally, other murine models have pointed to a tissue-protective role of IL-18, following injury to the intestinal epithelium. IL-18 may act via its cognate membrane receptor, IL18R1, that prevents the differentiation of CD4+ T helper 17 (Th17) and promoting the expression of key Foxp3+ regulatory T cell (Treg) effectors (Harrison et al., 2015; Mahapatro et al., 2021). Furthermore, studies have shown that inflammasome (NLRP6)-deficient mice, part of the inflammasome complex essential for processing of pro-IL-18 to its active form, showed reduced levels of IL-18 and alterations in the gut microbiota composition, including increased abundance of potential pathogens such as *Prevotella* and bacteria from the phylum *Saccharibacteria* (previously known as TM7) (Elinav et al., 2011; Levy et al., 2015; Chen, 2017). On the other hand, there is also data that support that IL-18 contributes to intestinal inflammation (Nowarski et al., 2015; Pu et al., 2019). Indeed, we observed increased levels of *IL18* in the inflamed intestinal mucosa of patients with IBD. Therefore, there appears to be a fine balance between the anti-inflammatory and pro-inflammatory actions of IL-18 in the intestinal mucosa. This equilibrium may be maintained through the direct influence of the soluble decoy receptor IL-18 binding protein (IL-18BP), which effectively neutralizes IL-18 activity *in vivo*, preventing excessive NF-κB activation and inflammation (Fantuzzi et al., 2003; Nowarski et al., 2015). Our findings indicate that mucosa-adherent bacteria, particularly *F. prausnitzii*, play a role in regulating *IL18* gene expression, primarily within the intestinal epithelium. This regulation may enhance barrier function and act as an anti-inflammatory factor, suggesting potential benefits for IBD patients in preventing disease flares.

*F. prausnitzii* plays an important role in maintaining healthy gut homeostasis, which is often linked to its capacity to produce short-chain fatty acids (SCFA), in particularly butyrate (Sokol et al., 2008; Cao et al., 2014; Laval et al., 2015; Moens and de Vuyst, 2017). Indeed, prophylactic *F. prausnitzii* administration reduced colonic paracellular permeability and neutrophil infiltration in a rat model of pelvic radiation disease, which was attributed to *F. prausnitzii*-mediated induction of intestinal epithelial IL-18 expression and secretion (Lapiere et al., 2020). In line, *IL18* gene and protein expression *in vitro* and *in vivo* have been shown to be increased by treatment of mice with SCFA or niacin (Kalina et al., 2002; Singh et al., 2014). Also, a positive significant correlation between *F. prausnitzii* abundance and mRNA and protein expression of NLRP6 was found in rats in an induced model for irritable bowel syndrome (IBS) (Bao et al., 2019). Similarly, activity of the NLRP3-containing inflammasome is induced in mice fed a fiber-rich diet and induced the production of IL-18 through the activation of intestinal epithelial SCFA receptors (GPR43 and GPR109A) (Macia et al., 2015). All these studies in animal models support our findings in IBD patients that *F. prausnitzii* abundance positively correlates to mucosal *IL18* gene expression, which is very likely maneuvered via activation of the HIF1α pathway. We thus provided first evidence of the interrelationship between butyrate-producing bacteria and HIF1α-dependent intestinal *IL18* expression in humans. We have, however, not yet identified butyrate *per se* as the *F. prausnitzii*-derived factor that induces *IL18* in the intestinal epithelium. However, we have established earlier that *F. prausnitzii* effectively produces butyrate when co-cultured with Caco-2 cells in the HoxBan system (Sadaghian Sadabad et al., 2015) and others have shown that butyrate activates HIF1α pathway in Caco-2 cells (Kelly et al., 2015; Zheng et al., 2017). Still, further studies are needed to fully understand this interrelationship.

We propose that the increase in gene expression of both *HIF1A* and *IL18* in inflamed biopsies is primarily driven by pathophysiological hypoxia, which is installed during inflammation in the intestinal mucosa (Fagundes and Taylor, 2017; Brown et al., 2020; Fagundes et al., 2022). Nonetheless, activation of the HIF1α pathway has been mechanistically linked to gut mucosal health, both in the context of prevention and recovery from intestinal inflammation. Blocking HIF1α in mouse models, especially on intestinal epithelial cells, impairs epithelial barrier integrity, because of dysregulation of HIF1α targets, such as tight junction proteins, intestinal trefoil factors and beta-defensins (Furuta et al., 2001; Synnestvedt et al., 2002; Kelly et al., 2013; Saeedi et al., 2015). Moreover, conditional knockout of HIF1α exacerbated inflammation in TNBS-induced colitis in mice, while increased HIF1α activation via knock-out of the von Hippel–Lindau (*VHL*) gene was protective (Karhausen et al., 2004). In our study, we show that stable knockout of *HIF1A* in Caco-2 cells decreased the expression of several well-known HIF1α target genes upon coculture with *F. prausnitzii*. Additionally, this led to suppression of 6 Gene Ontology biological processes, all involving the HIF1α-dependent regulation of *IL18*. In line, we detected 2 hypoxia-responsive elements (HRE) in the IL-18 promoter region, located 548 and 1,218 base pairs upstream of the first exon (Supplementary Figure S1). To the best of our knowledge, this is the first study to show HIF1α-mediated regulation of *IL18*, which may contribute to the protective role of HIF1α in the intestine.

Pharmacological activation of the HIF1α pathway promotes mucosal regeneration and intestinal barrier function in several mouse models of colitis (Cummins et al., 2008; Robinson et al., 2008; Gupta et al., 2014), as recently reviewed by Colgan & Taylor (Colgan et al., 2020). Prolyl-hydroxylase inhibitors (PHI) are a class of HIF-agonistic drugs that promote HIF1α activation by blocking the PHD-mediated degradation of HIF1α protein (Joharapurkar et al., 2018). Some of these drugs have already entered phase 2 and 3 clinical trials for the treatments of anemia of chronical renal disease, being well-tolerated by patients and healthy individuals (Haase, 2017). Thus, we postulate that PHIs may also be beneficial for IBD patients suffering from disease flares. Moreover, a beneficial effect may be expected from strategies to enhance the abundance of *F. prausnitzii* in the gut, thereby strengthening the fine epithelial HIF1α-*IL18* crosstalk. However, the applications of live *F. prausnitzii* as a probiotic therapeutic option remain a challenge, mostly because of its strict anaerobic nature. Alternatively, dietary fibers (prebiotics, such as inulin) could be used to increase *F. prausnitzii* abundance in the human gut (Verhoog et al., 2019). Although the anti-inflammatory effects of prebiotics in IBD patients remains controversial, co-treatment with PHI may be an interesting strategy, which would combine the regenerative potential of HIF1α activation with stimulation of growth of commensal bacteria.

In conclusion, our study reveals the interrelationship between *F. prausnitzii*, HIF1α activation and *IL18* expression in the intestinal mucosa. These factors are all relevant therapeutics to restore intestinal homeostasis in patients with IBD.

## Data availability statement

The patient-derived data from the Groningen 1000IBD cohort presented in this study are deposited in the European Genome-Phenome Archive (EGA) repository, with the accession number EGAS00001002702 (https://ega-archive.org/studies/EGAS00001002702; IDs: EGAD00001003991, EGAD00001008214, and EGAD00001008215). The transcriptomics data from HoxBan experiments presented in this study are deposited in National Library of Medicine (NHI) repository, with the accession number PRJNA954196 (https://www.ncbi.nlm.nih.gov/bioproject/954196; BioSample: SAMN34136424).

## Ethics statement

The studies involving humans were approved by the Institutional Review Board (IRB) of the University Medical Center Groningen (UMC Groningen) (in Dutch: “Medisch Ethische Toetsingscommissie,” METc; IRB no. 2008/338 and 2016/424). The studies were conducted in accordance with the local legislation and institutional requirements. The participants provided their written informed consent to participate in this study.

## Author contributions

RF: Conceptualization, Data curation, Formal analysis, Investigation, Methodology, Software, Visualization, Writing – original draft, Writing – review & editing. GB-R: Formal analysis, Methodology, Writing – original draft, Writing – review & editing. SH: Data curation, Formal analysis, Software, Visualization, Writing – review & editing. SK: Methodology, Validation, Writing – review & editing. RW: Conceptualization, Funding acquisition, Project administration, Resources, Software, Supervision, Writing – review & editing. CT: Conceptualization, Investigation, Supervision, Writing – original draft, Writing – review & editing. GD: Conceptualization, Funding acquisition, Investigation, Resources, Supervision, Writing – original draft, Writing – review & editing. HH: Conceptualization, Formal analysis, Funding acquisition, Investigation, Methodology, Resources, Supervision, Writing – original draft, Writing – review & editing. KF: Conceptualization, Formal analysis, Funding acquisition, Investigation, Project administration, Resources, Supervision, Writing – original draft, Writing – review & editing.
