# Supplementary material for: Faecalibacterium prausnitzii promotes intestinal epithelial IL-18 production through activation of the HIF1α pathway
Source: Front Microbiol. 2023 Dec 14;14:1298304. doi: 10.3389/fmicb.2023.1298304 (PMC10755969; doi:10.3389/fmicb.2023.1298304)
Supplement: Supplementary file 1 [file Data_Sheet_1.docx]

Supplementary Material

# Supplementary Figures and Tables

## Supplementary Figure


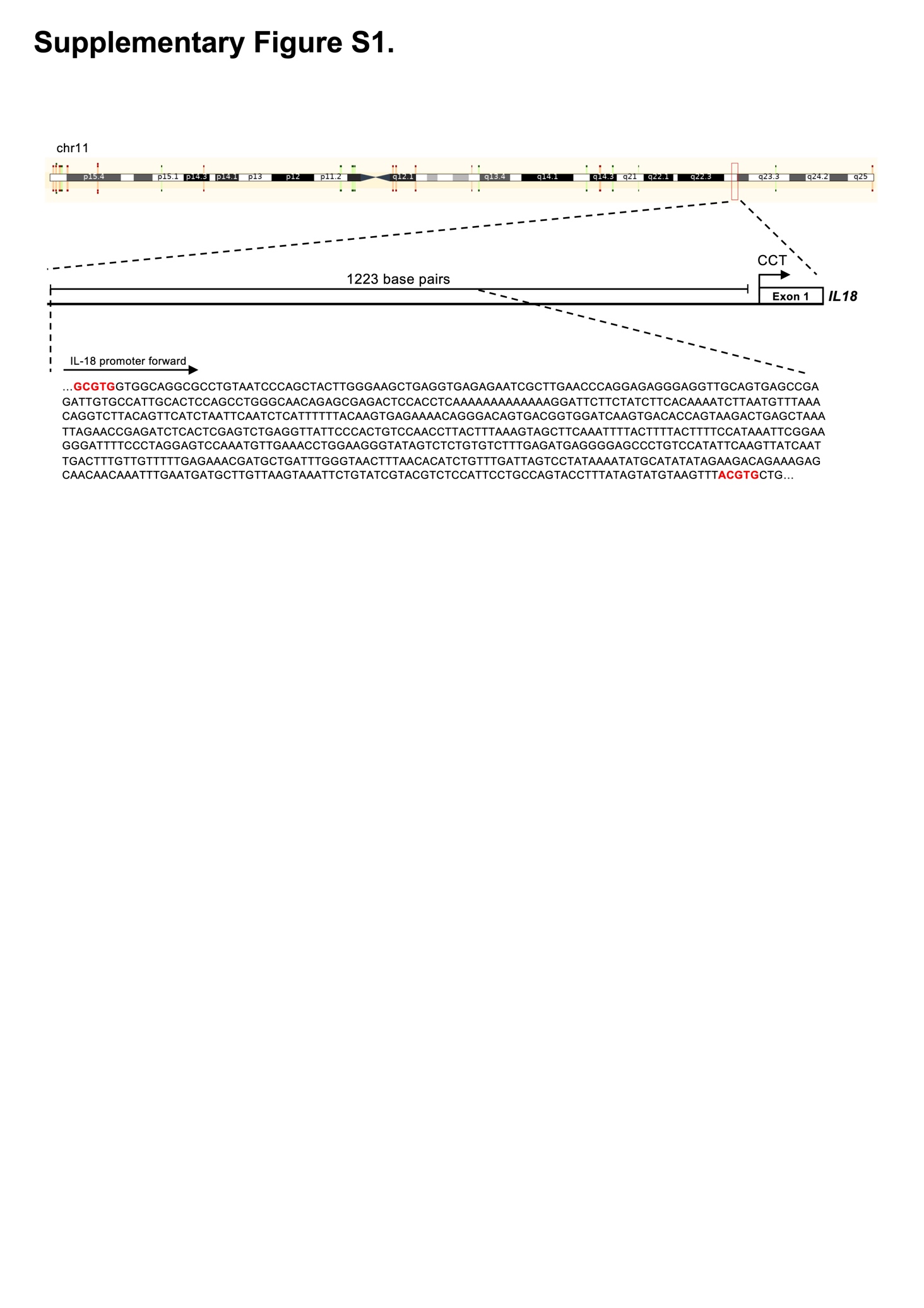


**Supplementary Figure S1**. Illustration of HIF1α binding site (hypoxia-responsive element; HRE) on *IL18* promoter and binding sites motives, HIF1α recognition sequence highlighted in red (not to scale).

## Supplementary Table

| **Supplementary Table S1. Sequences of probes and primers used for quantitative RT-PCR.** | | | |
| --- | --- | --- | --- |
| **Gene** | **Organism** | **Sequence** | |
| *18S* | Human | Probe | 5'-CGC GCA AAT TAC CCA CTC CCG A-3' |
|  |  | Sense | 5'-CGG CTA CCA CAT CCA AGG A-3' |
|  |  | Anti-sense | 5'-CCA ATT ACA GGG CCT CGA AA-3‘ |
| *PGK1* | Human | Probe | 5’- AGGCTGCTGTCCCAAGCATCAAATTCT-3’ |
|  |  | Sense | 5’- GTCGTTATGAGAGTCGACTTCAATG-3’ |
|  |  | Anti-sense | 5’-CGGCCTAGGTGGCTCATAAG-3’ |
| *HIF1A* | Human | Probe | 5-TTGCACTGCACAGGCCACATTCAC-3’ |
|  |  | Sense | 5’-TGAACATAAAGTCTGCAACATGGA-3’ |
|  |  | Anti-sense | 5’-TGAGGTTGGTTACTGTTGGTATCATATA-3’ |
| *EGLN3* | Human | Probe | 5’- TTCCTGAATTTCTTTTTGGCTTCTGCCCTT-3 |
|  |  | Sense | 5’-GACTGTCTGGTACTTTGATGCTGAAG-3’ |
|  |  | Anti-sense | 5’-GAGGGCAGATTCAGTTTTCCTAGTT-3’ |
| *IL18* | Human | Probe | 5’-TTCTGACTGTAGAGATAATGCACCCCGGAC-3’ |
|  |  | Sense | 5’-CCAAGGAAATCGGCCTCTATT-3’ |
|  |  | Anti-sense | 5’-CTTCACAGAGATAGTTACAGCCATACCT-3’ |

| **Supplementary Table S2. Bacterial genera correlated with *F. prausnitzii* abundance and *IL18* expression in intestinal mucosa of IBD patients.** | | | |
| --- | --- | --- | --- |
| **Bacteria (genera)** | **Effect size** | ***P*-value** |  |
| Positive associations: |  |  |  |
| *Ruminococcaceae_UCG.005* | 0.378 | 3.73×10^-27^ |  |
| *Agathobacter* | 0.320 | 1.84×10^-19^ |  |
| *Blautia* | 0.364 | 3.45×10^-25^ |  |
| Negative associations: |  |  |  |
| *Escherichia/shigella* | -0.251 | 2.29×10^-12^ |  |
